# Allograft dermal matrix hiatoplasty during laparoscopic primary fundoplication, paraesophageal hernia repair, and reoperation for failed hiatal hernia repair

**DOI:** 10.1007/s00464-012-2700-y

**Published:** 2013-01-09

**Authors:** Reginald C. W. Bell, Jacqueline Fearon, Katherine D. Freeman

**Affiliations:** SurgOne P.C., Swedish Medical Center, 401 W Hampden Place Suite 230, Englewood, CO 80110 USA

**Keywords:** Allograft, Acellular dermal matrix, Biologic mesh, Biomesh, Biologic prosthetic, Hiatoplasty, Hiatal hernia repair, Laparoscopic paraesophageal hernia repair, Routine mesh laparoscopic fundoplication

## Abstract

**Background:**

Hiatal repair failure is the nemesis of laparoscopic paraesophageal hernia repair as well as the major cause of failure of primary fundoplication and reoperation on the hiatus. Biologic prosthetics offer the promise of reinforcing the repair without risks associated with permanent prosthetics.

**Design:**

Retrospective evaluation of safety and relative efficacy of laparoscopic hiatal hernia repair using an allograft (acellular dermal matrix) onlay. Patients with symptomatic failures underwent endoscopic or radiographic assessment of hiatal status.

**Results:**

Greater than 6-month follow-up was available for 252 of 450 consecutive patients undergoing laparoscopic allograft-reinforced hiatal hernia repair between January 2007 and March 2011. No erosions, strictures, or persisting dysphagia were encountered. Adhesions were minimal in cases where reoperation was required. Failure of the hiatal repair at median 18 months (6–51 months) was significantly (*p* < 0.005) different between groups: group A (primary fundoplication with axial hernia ≤ 2 cm), 3.7 %; group B (primary fundoplication with axial hernia 2–5 cm), 7.1 %; group G (giant/paraesophageal), 8.8 %; group R (reoperative), 23.4 %. Additionally, mean time to failure was significantly shorter in group R (247 days) compared with the other groups (462–489 days).

**Conclusions:**

Use of allograft reinforcement to the hiatus is safe at 18 months median follow-up. Reoperations had a significantly higher failure rate and shorter time to failure than the other groups despite allograft, suggesting that primary repairs require utmost attention and that additional techniques may be needed in reoperations. Patients with hiatal hernias >2 cm axially had a recurrence rate equal to that of patients undergoing paraesophageal hiatal hernia repair, and should be treated similarly.

Recurrence of hiatal hernia is the nemesis of treatment of giant or paraesophageal hernias [1, 2]. It is also the major reason for failure after laparoscopic primary fundoplication [3–6] and, though not as well documented, the most common reason for failure after reoperation on the esophageal hiatus [7, 8]. Although this issue has been recognized for some time, debate still continues as to whether prosthetic reinforcement of the hiatus has enough benefit to offset potential risks of erosion or adhesion [9–12].

Following reports of short-term benefit to collagenous prosthetic reinforcement of the hiatus [13], in 2007 we began using an allograft dermal matrix (Allomax; Davol, Inc., Warwick, RI) as an onlay after primary suture repair of giant or paraesophageal hernias. The experience was then extended to using this prosthetic in recurrent hiatal hernias, in line with surgical principles learned from repairing recurrent ventral and groin hernias. Extension of this concept brought us to consider the use of an allograft onlay after repair of all hiatal hernias during primary fundoplication. This study is a retrospective analysis of our experience in using a single type of allograft dermal matrix in all patients having laparoscopic hiatal hernioplasty during primary fundoplication, repair of giant/paraesophageal hernias, or revisional surgery.

## Materials and methods

### Study population

Beginning in January 2007, all patients undergoing laparoscopic hiatal hernia repair (whether as part of a fundoplication for reflux control, or repair of a giant hiatal hernia, or reoperation for failed prior surgery) were reinforced with a dermal allograft onlay when primary hiatal closure could be performed. This patient population had been followed clinically and represents the cohort for this retrospective study.

### Study design and endpoints

This retrospective study was approved by the HealthOne institutional review board (IRB) and is registered at irbnet.org (IRBNet ID: 231822-3). As a retrospective study of a clinical database, informed consent was not required.

#### Treatment

The treatment employed for the study was reinforcement of primary closure of the hiatus using an allograft dermal matrix (Allomax; Davol, Inc.). This allograft is terminally sterilized human dermal collagen of 0.8 mm to 1.8 mm thickness. Using a proprietary process, all noncollagenous cellular components are removed but constituent elastin fibers remain. The allograft is not cross-linked.

#### Patient stratification

Patients were stratified into four groups. Group A consisted of patients presenting with an axial hiatal hernia of ≤2 cm. Group B patients had a presenting axial hiatal hernia >2 cm but less than 6 cm, without significant paraesophageal component. Group G (giant) was formed of patients with a giant or paraesophageal hiatal hernia, regardless of the axial height of the hernia. Group R (redo) was formed of patients undergoing reoperation for a failed prior procedure on the hiatus or gastroesophageal (GE) junction, regardless of the size of the hernia.

### Safety

Adverse events were defined as dysphagia requiring dilation in the first 3 postoperative months, dysphagia requiring reoperation, mesh erosion or infection. In addition, density of adhesions at reoperation was recorded.

### Endpoints

Subjective outcomes were assessed by patient self-reported assessment of the success or failure of the procedure. Clinical failure was defined as any recurrence or development of troublesome typical GE reflux disease (GERD) symptoms more than 2–3 times per week.

Patients who reported clinical failure were asked to undergo objective evaluation to determine if recurrent reflux or recurrent hiatal hernia was present. Objective outcomes were assessed using esophagogastroduodenoscopy (EGD) and/or barium swallow. Failure of the hiatal repair was defined as >2 cm axial component to hiatal hernia, or any degree of paraesophageal herniation or transthoracic wrap migration, by either study.

#### Preoperative assessment

All patients undergoing surgery had objective evidence of GERD, objective evidence of a giant hiatal hernia, or postfundoplication symptoms severe enough to warrant reoperation (e.g., dysphagia). Patients having surgery for GERD had medically refractory GERD, desired an alternative to medical therapy, or had laryngopharyngeal symptoms thought likely due to GERD. The great majority of GERD patients had preoperative ambulatory reflux testing, endoscopy, and esophageal manometry. Patients having surgery for a giant or paraesophageal hiatal hernia had preoperative endoscopy and, when indicated, barium swallow or esophageal manometry. Patients undergoing reoperation had preoperative endoscopy, ambulatory reflux testing, and manometry whenever possible. Solid gastric emptying studies were obtained selectively. Informed consent included the use of a prosthetic reinforcement of the hiatal closure.

### Technique

During laparoscopic surgery on the hiatus, following dissection of the phrenoesophageal membrane and excision of the hernia sac (if present), mediastinal mobilization of the esophagus was performed until adequate (2–3 cm) intraabdominal esophagus was obtained. Evaluation of the location of the angle of His both laparoscopically and by the gastroesophageal junction (or end of the tubular esophagus) was used. If adequate length could not be obtained, a Collis gastroplasty was performed with a single endoscopic linear staple line, the stapler being introduced through the left chest.

Once adequate esophagus or neoesophagus was obtained to permit a tensionless fundoplication, the diaphragm hiatus was closed using nonabsorbable polyester suture (0-Ethibond; Ethicon, Inc.). Sutures were placed primarily posteriorly; however, anterior and/or left-sided figure-of-eight sutures were placed when needed to close the hiatus. Hiatal orifice following closure was between 2.5 and 3 cm diameter without a bougie, which is equivalent to precise closure around a 60-Fr bougie.

**Fig. 1:**
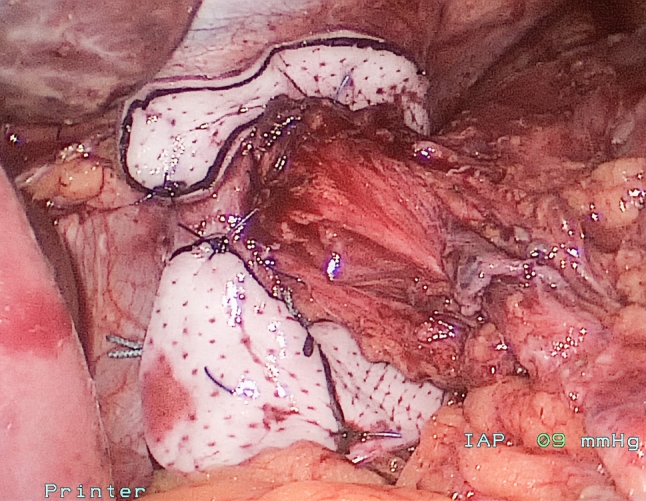
Operative photograph of allograft onlay

Once primary closure was accomplished, an allograft dermal matrix (Allomax; Davol Inc.) was placed as an onlay over the repair. Initially, the allograft was placed just over the posterior closure as a heart-shaped patch; gradually, the shape of the patch evolved to a “U”- and then to a “C”-shaped patch. The patch was secured in place with three or four absorbable monofilament 3-0 sutures at the edges of the hiatus and to the dorsal aspect of the crural closure; further sutures were placed at the far edges of the patch to distribute tension when judged clinically necessary (Fig. 1). Care was taken that the patch lay smoothly against the peritoneal (defatted) surface of the diaphragm. Initially the patch was placed with the dermal side against the diaphragm; beginning in 2009, as a result of anecdotal feedback from plastic surgeons, the patch was placed with the epidermal side toward the diaphragm. [If primary closure could not be achieved, a permanent polytetrafluoroethylene (PTFE) patch was used to complete the hiatal closure; these five patients are not part of this analysis.]

A fundoplication (Nissen or Toupet) was performed in all instances after the hiatal closure, even for paraesophageal hernias without reflux symptoms. The fundoplication was not secured to the crura; in the Toupet, two suspensory sutures were placed to the diaphragm 1 cm away from the hiatus at 11 and 1 o’clock (considering the hiatus as a clock face, viewed from below).

Antibiotics were not routinely administered.

### Postoperative care

Postoperative care involved commonly accepted practices of antiemetic medication and gradual reintroduction of thicker-consistency foods. Patients were instructed to avoid heavy lifting or vigorous exercise for 4 weeks after surgery.

### Follow-up assessments

Patients were contacted as part of our routine clinical practice in patients undergoing laparoscopic fundoplication or hiatal hernia repair. Intervals of follow-up varied as clinical practice permitted. Patients were asked to gauge the success of the surgery. Patients who met criteria for clinical failure were asked to undergo objective evaluation to determine the cause of failure. Additionally, any patient who had troublesome dysphagia or chest pain was asked to undergo objective evaluation.

### Data analysis

Data were entered into an electronic database system (Microsoft Access) and monitored for accuracy against the source documents. Aggregate data with patient demographics, baseline characteristics, efficacy, safety, and patient satisfaction results were summarized by descriptive statistics. Mean, standard deviation (SD), and standard error of mean (SEM) were generally reported for continuous variables. Median and range were reported for data with skewed distribution. *p*-Values for changes at follow-up compared with baseline were calculated using the Mann–Whitney *U* and paired *t* test. Fisher’s exact test was used to compare frequencies. Multiple groups were compared using analysis of variance (ANOVA) or Kruskal–Wallis test. Values with *p* < 0.05 were considered significant. All analyses were performed using XL Stat software.

## Results

### Patient characteristics at baseline

Between 1 January 2007 and 31 May 2011, 400 patients underwent laparoscopic hiatal hernia repair with reinforcement using the allograft. Attempts by phone, email, and letter resulted in 252 patients available for follow-up. Baseline characteristics of the entire study population as well as stratification of the four groups are presented in Table 1.

**Table 1:** Patient characteristics at baseline

	Total	Group A (≤2 cm)	Group B (2–5 cm)	Giant	Redo
Number	252	81	54	67	50
M/F	89/166	28/53	22/32	17/50	20/30
BMI (mean, SD), kg/m^2^	30 (5.7), range 49–18	30.6 (6.1)	30.1 (5.1)	29.5(5.5)	29.7 (6)
Age, years	57 (13.4)	52 (12)	58 (13)	63 (12)	55 (14)

Of the 252 patients, 244 were on proton pump inhibitor (PPI) therapy at presentation, only 10 % reported ≥90 % response of their primary symptom to PPI therapy, and median response to PPI therapy was 25 %.

**Fig. 2:**
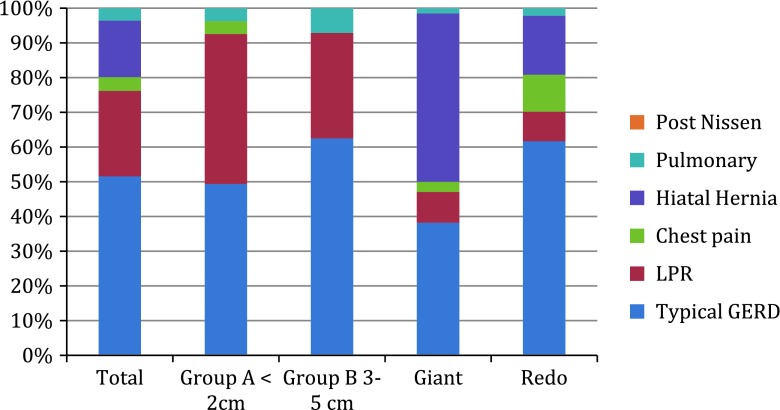
Category of presenting symptoms per group. *LPR* LaryngoPharyngeal Reflux

Category of presenting primary symptoms per group is presented in Fig. 2.

All patients had objective evidence of GERD or a concerning giant/paraesophageal hiatal hernia prior to surgery. Objective evidence included abnormal ambulatory reflux monitoring, erosive esophagitis >LA class A, columnar-lined epithelium (CLE) with or without specialized intestinal metaplasia (SIM), or a large fixed or paraesophageal hiatal hernia with chest pain, dysphagia, or evidence of organoaxial volvulus.

Group A [axial hiatal hernia (HH) ≤ 2 cm] consisted of 81 patients, and group B consisted of 56 patients (axial HH >2 to <6 cm), undergoing primary fundoplication for GERD. Group G consisted of 68 patients with axial hiatal hernias of at least 6 cm, or paraesophageal hernias, undergoing primary surgery. All of these patients underwent a fundoplication after the hernia repair. Group R consisted of 47 patient undergoing revisional surgery for a failed prior fundoplication or prior hiatal hernia repair (35 with one prior surgery, 7 with two prior surgeries, and 3 with three prior surgeries). The precise reasons for the primary surgery in these patients, e.g., primary GERD or paraesophageal hernia, were frequently not available even after review of the original operative reports, but for the most part appeared to be primary GERD. Operative details are reviewed in Table 2.

**Table 2:** Operative findings

	Total	A	B	G	R	*p*
*N*	252	81	56	68	47	
Hiatal hernia height (cm) (mean)	2.3	1.2	2.4	3.8	1.7	<0.0001
Hiatal width (cm) (mean)	2.75	2.4	2.7	3.1	3.0	<0.0001
Hiatal AP (cm) (mean)	5.3	4.8	5.4	6.1	5.3	<0.0001
Extended mediastinal mobilization >5 cm (%)	54 %	20 %	45 %	94 %	60 %	<0.0001
Collis (%)	8 %	0 %	2 %	27 %	4 %	<0.0001
Toupet (%)	11 %	25 %	9 %	0 %	6 %	<0.0001
No. posterior sutures (median, range)	3 (0–5)	2 (1–4)	3 (1–4)	3 (1–5)	3 (0–4)	
With anterior sutures (%)	49 %	26 %	52 %	67 %	46 %	<0.0001
With left sutures (%)	13 %	0	1 %	12 %	23 %	
Heart-shaped Allomax (%)	24	29	23	19	22	
U-shaped Allomax (%)	21	18	20	15	32	
C-shaped allograft (%)	55	53	57	66	46	
No. heart-shaped allografts (failures)	60 (4)	25 (1)	13 (1)	13 (1)	9 (1)	ns
No. U-shaped allografts	52 (8)	14 (0)	11 (1)	11 (3)	16 (5)	0.08
No. C-shaped allografts	140 (11)	42 (2)	32 (2)	45 (2)	22 (5)	0.057

As would be expected from the initial patient grouping, hiatal characteristics varied between groups. The giant hiatal hernia group required more extensive mediastinal mobilization, and 27 % received Collis gastroplasty. Both giant and redo groups had more anterior and left crural closure sutures placed than the primary groups (A and B).

### Procedure and safety outcomes

Three patients had postoperative perigastric infections, all within the first 20 days of surgery; all were in the reoperation group. In these three patients the infection resolved without removal of the allograft.

Fourteen patients (5.6 %) required more than one dilation in the first 90 days after surgery. These patients typically evidenced resistance to passage of a Maloney dilator at initial dilation; serial radial and bougie dilations to 18 mm (generally three procedures) resulted in loss of resistance and relief of dysphagia in all five. Although a greater percentage of group A required dilation (8.6 % versus 1.8–4.3 %), this did not reach statistical significance (*p* = 0.2, Chi square). There were three instances of hiatal stenosis that underwent laparoscopic release after failed dilations (one, three, and three). The hiatus was simply incised on the right anterior aspect sufficiently to admit a 56-Fr Maloney dilator without resistance. These patients were early in our series, and all experienced immediate relief of the dysphagia and ability to eat a solid diet within 1 week of the release.

No instances of allograft erosion were observed in this series, nor have any been observed in more than 450 patients operated on to date.

Patients who came for reoperation with prior allograft placement did not evidence significant or difficult adhesions. In fact, the solitary permanent braided polymer suture that was used to secure the mesh generated significantly denser adhesions than the allograft. An empirical observation is also that tissue sealants/glue generate much greater difficulty during reoperation than does the allograft.

### Outcomes

All patients had follow-up >180 days with the exception of five early failures going to reoperation; mean follow-up was 17.7 months (6–51 months). No significant difference in follow-up duration existed between the four groups.

**Fig. 3:**
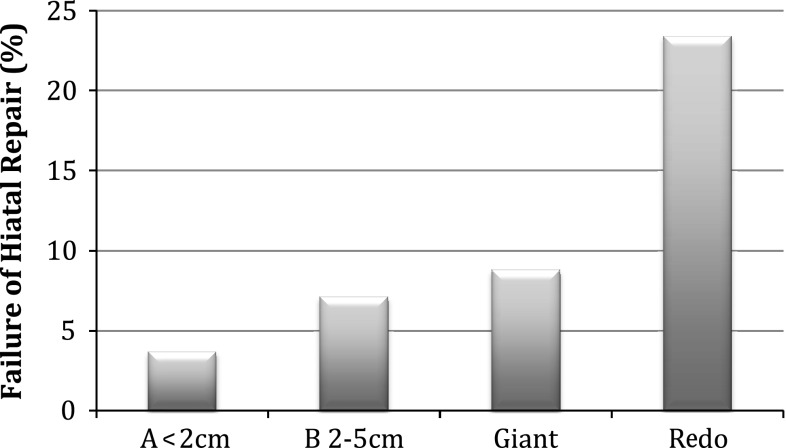
Hiatal failure rates at 18-month median follow-up

Failure of hiatal repair was defined as development of esophageal symptoms with objective evidence of transthoracic migration of the wrap or the stomach. Overall, 24 patients (9.5 %) evidenced symptomatic failure due to recurrent hiatal hernia. (All patients with subjective recurrence agreed to objective testing to determine their hiatal anatomy.) Group A (≤2 cm) developed symptomatic hiatal failure in 3.7 % of cases, whereas the 2–5-cm axial hernias (group B) and the giant hiatal hernia groups failed at a similar rate (7.1 % and 8.8 %). The reoperative group (R) failed at a significantly greater rate of 23.4 % at 18 months (*p* < 0.003, Chi square) (Fig. 3). No symptomatic failures possessed preoperative body mass index (BMI) >40 kg/m^2^. (Twelve patients possessed preoperative BMI >40 kg/m^2^; five were >45 kg/m^2^.) Four failures occurred in 41 patients with preoperative BMI >35 kg/m^2^ (10 %), no different from the overall group. Numbers of patients with various BMI in the subgroups were too small to draw any conclusions.

**Fig. 4:**
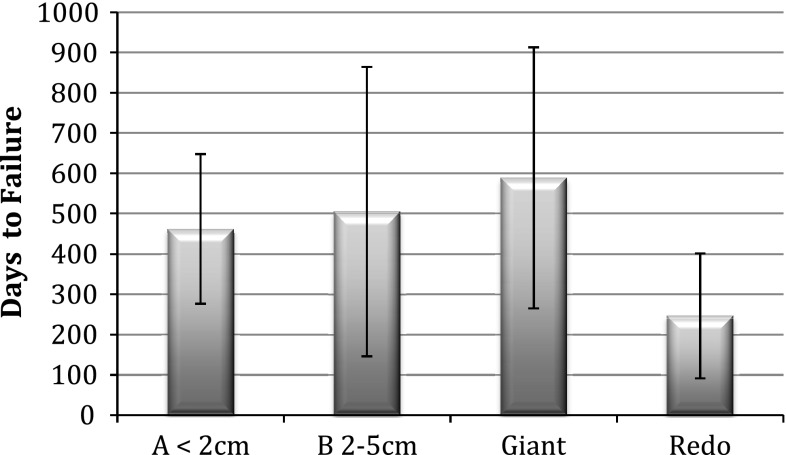
Time to hiatal failure per group (mean, SD)

Time to failure was also analyzed, and again the reoperative group failed significantly earlier (mean 247 days) compared with the three other groups (462–589 days) (Fig. 4).

Neither the extent of mediastinal mobilization, the number or location of sutures, the addition of Collis gastroplasty, nor the mesh shape (heart, U, or C) affected risk of recurrence.

## Discussion

The most common cause of failure after *primary* laparoscopic fundoplication done for GERD is hiatal failure, accounting for up to 90 % of failures in some series [3, 5, 14, 15]. Failure after primary suture repair of giant/paraesophageal hiatal hernias is determined by failure of the hiatoplasty, and rates approach 25 % at 3 years in many studies [2, 16], though various methods of defining failure have been used. The specific reasons for failure after revisional surgery have infrequently been reported [17].

Since the 1980 s, operative technique for inguinal and ventral hernia repair has undergone a paradigm shift: from prosthetic rarely used, to prosthetic use for recurrent hernias, to prosthetic use for large hernias, to prosthetic use even for routine smaller hernias. The same shift to prosthetic use cannot be said for hiatal hernia repair, and debate continues on whether or not to use permanent or biologic prosthetics during paraesophageal hiatal hernia repair and, to a lesser extent, during primary laparoscopic fundoplication [11, 12]. This debate in part revolves around whether recurrence rates are reduced, and in part around whether erosion risks supersede any potential reduction in recurrence rates. Use of permanent prosthetic materials in patients undergoing primary fundoplication has been demonstrated to decrease recurrence rates compared with primary repair in randomized studies [18, 19]. However concerns about erosion or permanent dysphagia have limited the adoption of permanent prosthetics, not only in primary fundoplication but also in giant/paraesophageal hiatoplasty [20]. A 2006 review of published literature on permanent prosthetics in both primary fundoplication and paraesophageal hernia repairs found improved outcomes in the mesh group, with very rare instances of erosion reported. Interestingly, the authors state that their current practice for large paraesophageal hernias with closure under tension was to add an onlay acellular dermal matrix [21].

In 2006, Oeschlager [13] reported that a prospective, randomized study of porcine intestinal submucosa (Surgisis; Cook Medical) prosthetic reinforcement of paraesophageal hiatal hernias found a significant difference in radiographic recurrence rates at 6 months. Five-year follow-up however did not find a difference in radiographic or symptomatic results [22]. Jacobs [23] in 2007 reported the outcomes of 59 (of 92) patients who underwent hiatal hernia repair without mesh compared to 92 (of 127) patients who underwent hiatal hernia repair with porcine intestinal submucosa reinforcement. At median 3.2-year follow-up, symptomatic recurrence due to recurrent hiatal hernia occurred in 20 % of the nonmesh group compared with 3.2 % of the group reinforced with biologic graft. E. Lee [24] studied 17 patients undergoing paraesophageal hernia repair with allograft reinforcement and found two radiologic recurrences at median 10-month follow-up, one of which was symptomatic. Y. Lee [25] reported a 3.8 % radiologic and symptomatic recurrence rate in 52 patients with allograft (AlloDerm) reinforcement of large hiatal hernias at >1-year follow-up; however, it is not clear that all patients had radiologic follow-up. Wisbach [26] found 1 radiologic recurrence in 11 patients having paraesophageal hernia repair with allograft at mean 1-year follow-up. Diaz [27] found 2/46 symptomatic recurrences, and 2/26 radiologic recurrences, in patients undergoing paraesophageal hernia repair with allograft reinforcement (Allomax) with 3.6-month follow-up [27]. Of note, no erosions were observed in any of these studies. Persistent dysphagia was not reported in any of the studies other than that of Diaz (13 %), which had a relatively short follow-up.

We began in 2007 performing *routine* biologic prosthetic reinforcement of the hiatus in *all patients* undergoing surgery on the hiatus, because of our personal experience that 90 % of primary fundoplication failures, all paraesophageal hernia repair failures, and at least 90 % of reoperation failures involved hiatal failure. We were not content with these failures, even when overall failure rates for primary fundoplication were relatively low. Concern for erosion potential with permanent prosthetics led us to consider biologic materials. Reports that acellular human dermis (Alloderm) as well as porcine intestinal submucosa prosthetic reduced recurrences in giant hiatal hernia repair [28] were encouraging. We used one prosthetic (Allomax; Bard, Inc.). This is a non-cross-linked allograft that rehydrates quickly, does not require special storage, and may possess greater initial durability in comparison with porcine intestinal submucosa materials.

Our experience to date with placing this particular biologic prosthetic in over 450 patients has demonstrated no persistent dysphagia, no erosion, and minimally difficult adhesion formation at reoperation. This is in concert with a report from the University of Washington reporting no erosions, strictures, or other complications related to mesh use in 73 of 126 patients followed for a median of 45 months after laparoscopic paraesophageal hernia repair with onlay biologic reinforcement [29].

Some patients in our series experienced early postoperative dysphagia severe enough to require dilation. During dilation, the use of Maloney dilators was accompanied by resistance that lessened with serial dilations, indicating that hiatal fibrosis and narrowing was the likely cause. Others have not reported problems with dysphagia, perhaps related to different placement of the biologic patch [30]. Having observed recurrences on the left and anterior aspect of the hiatus, we have come to prefer a reverse-C-shaped patch attached fairly close to the hiatal opening.

We are unaware of other studies examining differences in hernia recurrence rates between patients undergoing primary fundoplication, giant hiatal hernia repair, and revisional surgery, especially utilizing a dermal allograft. This study demonstrated a significant difference in both hiatal hernia recurrence rates and time to recurrence between primary fundoplication and reoperative surgery on the hiatus. Additionally, failure rates of patients with hiatal hernias >2 cm were similar to patients with giant hiatal hernias.

Because the current study lacks a control arm of patients without prosthetic reinforcement, we cannot draw definitive conclusions regarding whether or not the addition of a biologic prosthetic decreases recurrence rates. It may be that biologic grafts delay rather than prevent recurrence [22]. Delay may be a reasonable outcome, however, as reoperation is associated with an even higher risk of recurrence. Safely preventing recurrence in a situation in which the esophagus passes through a muscular opening, with continual movement and dilation of the esophagus, may be an unrealistic goal with current techniques. Perhaps re-creation of a viable phrenoesophageal membrane/ligament, i.e., restoration of normal anatomic constructs, will be needed in addition to reapproximating the hiatus.

This study using biologic prosthetic in all patients undergoing hiatal repair regardless of size found that symptomatic recurrence rates were similar between patients with a hiatal hernia between 2 and 5 cm (group B) and patients with giant hiatal hernias (≥6 cm axial height). This, combined with the finding of an extremely high (23 %) hiatal failure rate in reoperative cases despite biologic prosthetics, suggests (but does not demonstrate) (1) that maximal therapy be used at the first operation, (2) that whatever benefit biologics confer to giant hiatal hernia repair may extend similarly to the repair of lesser hiatal hernias of >2 cm axial height, and (3) that in reoperations, addition of biologic prosthetics alone may not be an adequate solution.

This study is limited by (1) its retrospective nature with incomplete follow-up (63 %, 252/400), (2) the use of clinical criteria to define failure or success and evaluating only clinical failures for objective evidence of hiatal hernia recurrence, and (3) the lack of a control arm of patients not undergoing prosthetic reinforcement. (1) With regards to follow-up, we have found that patients with recurrent problems tend to be referred back to us and not to other centers, and we believe that the 252 patients completing follow-up are fairly representative of the entire group. (2) The primary endpoint was clinical failure that was confirmed objectively to be due to hiatal failure. Although patients had various presenting symptoms, any recurrence of primary symptoms or development of de novo symptoms that could indicate recurrence was considered a clinical failure. All such clinical failures were evaluated objectively by barium swallow or endoscopy and determined to be objective failures if a hiatal hernia was found. This approach is in concert with the clinical situation that patients and practitioners face: in a patient with recurrent symptoms, how often is that recurrence due to hiatal failure? Other studies using radiographic recurrence as a primary endpoint have demonstrated that many radiographic recurrences are asymptomatic and do not require reoperation [31]. (3) Although the study lacks a control arm of patients not having a prosthetic, this study compared outcomes among four different groups treated in the same manner. Patients with >2 but <6 cm axial hernias were found to have a similar rate of symptomatic recurrence after hiatal hernia repair as did patients with giant/paraesophageal hiatal hernias. Patients undergoing reoperative surgery demonstrated a *significantly* greater hernia recurrence rate, and shorter time to recurrence, than patients undergoing primary fundoplication. We believe the comparative findings of (a) similar recurrence rates between patients with >2 cm axial hernias and patients with giant hiatal hernias when an allograft was used in both groups, and of (b) significantly worse outcomes in reoperations despite use of a biologic prosthetic, are the most important results of the current study.

## Conclusions

This retrospective analysis of 252 patients undergoing hiatal hernia repair reinforcement with a dermal matrix allograft found the procedure to be safe, with a low incidence of postoperative dysphagia and no issues with erosion or dense adhesion formation. Additionally, at 18-month median follow-up, patients undergoing reoperation had very high (23 %) recurrence rates and shorter time to failure despite allograft use. Patients with hiatal hernias between 2 and 5 cm axial height demonstrated failure rates comparable to patients with giant hiatal hernias. It is unclear whether patients with hernias of ≤2 cm axial dimension benefit from prosthetic reinforcement. Patients undergoing reoperation probably require more than allograft reinforcement.
